# Prostaglandin-induced cervical remodelling in humans in the first trimester is associated with increased expression of specific tight junction, but not gap junction proteins

**DOI:** 10.1186/1479-5876-10-40

**Published:** 2012-03-07

**Authors:** Vidita V Ghulé, Colin Gray, Andrea Galimberti, Dilly OC Anumba

**Affiliations:** 1 Academic Unit of Reproductive and Developmental Medicine, University of Sheffield, Sheffield, UK; 2 Department of Cardiovascular Science, University of Sheffield, Sheffield, UK; 3 Academic Unit of Reproductive and Developmental Medicine, Level-IV, The Jessop Wing, University of Sheffield Medical School, Tree Root Walk, Sheffield S10 2SF, UK

**Keywords:** Cervical remodelling, Gap junctions, Prostaglandins, Tight junctions, Misoprostol, Pregnancy

## Abstract

**Background:**

Prostaglandins (PG) are widely employed to induce cervical remodelling (CR) in pregnancy. However, the underlying molecular mechanisms are not fully elucidated. Tight junctions (TJ) and gap junctions (GJ) regulate paracellular and intercellular solute transport respectively but their role in the process of CR remains unexplored. We hypothesized that the synthetic prostaglandin E1 analogue Misoprostol (M), widely used in clinical practice to induce CR, may alter TJ and GJ expression as part of the changes in the extracellular matrix (ECM) associated with remodelling. We investigated the effects of Misoprostol exposure on the expression of cervical TJ (claudins 1, 2, 4, 5, 7 and occludin) and GJ (connexins 43, 30 and 26) in the 1st trimester.

**Methods:**

Cervical biopsies were obtained from pregnant women and comparisons of TJ and GJ protein expression (by western blotting) and immunolocalisation (laser scanning confocal microscopy) made between those who were administered vaginal Misoprostol (n = 10) and those who were not (n = 5).

**Results:**

We found that Misoprostol-treated tissue (M+) had higher expression of Claudins 1,2,4,7 and occludin (p < 0.05) than untreated (M-) tissue. Expression levels of Claudins 1, 2 and 4 were positively correlated to interval from Misoprostol treatment to biopsy, whilst occludin was negatively correlated. Misoprostol-treated cervical tissue demonstrated more endothelial claudin-5 and occludin, whilst expression of GJs were unchanged.

**Conclusion:**

Our observations suggest, for the first time, that increased expression of tight junction proteins may be one of the mechanisms by which Misoprostol induces CR in humans. Further studies are needed to explore if TJ proteins may be therapeutic targets to alter timing of CR in clinical practice.

## Background

The mechanisms by which the rigid tubular cervix softens, thins out and dilates (cervical remodelling, CR) during pregnancy and before parturition are not fully understood. Cervical remodelling is pivotal to the clinical course and outcome of labour. Thus, understanding the underlying molecular mechanisms of the process may facilitate the prediction, prevention, or initiation of labour in clinical practice.

Connective tissue predominates the cervix and undergoes most of the remodelling of that organ. Although findings vary with experimental models and species, CR is associated with increased stromal hydration [1], disorganization of collagen bundles [2,3] and altered concentrations of hyaluronic acid and dermatan sulphate [4]. During the final stages of CR, there is increased nitric oxide synthesis [5], with extravasation of leucocytes [6] and activated fibroblasts [7]. A role for the epithelium during CR is suggested by its expression of cyclo-oxygenase (COX)-2, interleukin-8 (IL-8), glucocorticoid receptors [7-9] and aquaporin channels [10].

Prostaglandins (PGs) are widely used to ripen the pregnant cervix. Easier mechanical dilation of the softer 'PG-primed' cervix has significantly reduced operative morbidity associated with surgical termination of pregnancy (STOP) [11-13]. Misoprostol, a synthetic 15-deoxy 16-hydroxy 16-methyl analogue of natural PG-E1 has been the drug of choice due to its effectiveness, low cost and minimal side effects at a dose of 400 micrograms (μg) for 3 hours [11,14]. Human cervical biopsy studies in 1st trimester suggest that exogenous PG induces CR by causing disorganization of collagen, vasodilatation, increasing stromal leukocytes, and stimulating the release of pro-inflammatory cytokines [6,15,16]. However the mechanisms by which vaginally administered PG induces these changes in the extracellular matrix are not fully established.

TJs are sites of intercellular contact in the most apical regions of the lateral membranes of epithelial and endothelial cells, and regulate paracellular water and solute transport. TJs are composed of cytoplasmic scaffolding proteins (zona occludens) and tetra-span trans-membrane proteins such as junction adhesion molecules, occludin and claudins [17]. Non-pregnant human cervical ectothelium, which is stratified squamous variety, has been shown to express claudins 1, 2, 4, 7 and occludin [18,19]. Changes in transcripts of cervical claudins 1 and 2 have been described in pregnant mice [20]. As predominant regulators of paracellular water and solute transport [21], it is possible that changes in functional expression of TJs may contribute to CR. Animal studies have shown that TJ may modulate the permeability of cervical epithelium during pregnancy [20]. GJs are also trans-membrane channels connecting the cytoplasm of the adjacent cells. They facilitate free transfer of ions up to 1 kiloDalton (kDa) [22]. Expression of Gap Junction (GJ) proteins, namely Connexins (Cx) 43, 26 and 30 in human cervical ectothelium has been described [23,24], but whether they play a role in CR is unknown. It has been postulated that tissue specific co-expression of various GJ and TJ proteins, by virtue of their selective permeability, may regulate tissue adaptation to various physiological needs [22].

We hypothesized that the PGE1 analogue Misoprostol may alter cervical TJ, and perhaps GJ expression, as part of the process of CR in a time-dependent fashion.

## Methods

Two 3 × 3 × 10 mm biopsies were collected trans-vaginally from the anterior lip of the ectocervix from fifteen women prior to STOP under general anaesthetic between 9 and 11 (mean = 10.4) weeks of viable gestations not known to have fetal malformations with Trischler's punch biopsy forceps by a single trained researcher (VG). Ten women received 400 micrograms vaginal Misoprostol (M) with mean interval from treatment to biopsy of 95 min (range 50-140 min) while 5 women did not by choice. The mean ages of the treated and un-treated groups were 24.5 years (range 19-34) and 27 years (range 23-32) respectively. The North Sheffield research ethics committee approved this study (ref-08/H1310/35). All women gave written informed consent. Inclusion criteria were age between 18-45 years, previous vaginal delivery, and viable singleton pregnancy less than 12 weeks. Women with a history of previous cervical surgery, abnormal cervical smears, vaginal bleeding or cervico-vaginal infections within one week of the biopsy were excluded. We studied only parous women in order to: a) achieve some degree of homogeneity in the study groups, and b) ensure that normal cervical function and compliance had been demonstrated by previous successful vaginal delivery. Furthermore, all nulliparous women are given Misoprostol prior to STOP to facilitate surgical dilatation and would have been unsuitable for our studies since we also wanted to investigate cervical tissue not pre-treated with Misoprostol. All samples were studied by both Western blotting (WB) and Immunofluorescence (IF) and representative images and blots have been shown in the figures.

### Western blot analysis

The tissue was homogenized in 1 ml of ice cold RIPA (Radio-Immuno-Precipitation-Assay) buffer [1 M Tris hydrochloride (pH 7.4), 150 mM sodium chloride, 500 mM Ethylenediamine tetra-acetic acid with protease and phosphatase inhibitors (1 μl/ml each of Aprotinin, Leupeptin, Pepstatin, dithiothreitol)]; Phenylmethylsulfonylfluoride 10 μL/ml and sodium fluoride 5 μL/ml-(Roche Diagnostics, Germany) and detergents [Triton-X100-1 μl/ml, 10% Sodium Dodecyl sulphate (SDS)-10 μl/ml and sodium deoxycholate-25 μl/ml]. The supernatant was obtained by centrifugation at 15000 g for 20 min at 4°C and total protein estimated by Nano-Drop™ 1000 spectrophotometer (Thermo Scientific, UK) at absorbance 280 nm. Samples were prepared by adding an equal volume of SDS loading buffer [final volume of 48 ml: 6.0 ml 5 mM Tris (pH 6.8), 4.8 ml 100% (v/v) glycerol, 9.6 ml 10%SDS (v/v), 2.4 ml beta-mercaptoethanol, 1.2 ml 0.5% (w/v) bromophenol blue and 24 ml MQ water] to the homogenate. 100 μg of crude protein was loaded in 12.5% polyacrylamide gels. SDS PAGE was carried out in 1XSDS buffer (25 mM Tris, 192 mM Glycine, 0.1% SDS) at 75 V over 2 hrs in the Mini-PROTEAN tetra cell (Bio-Rad, UK). Following wet transfer on PVDF membrane (Millipore, USA) at 100 V for 75 min at 4°C, the blots were blocked in 5% non-fat milk in 1XPBS at room temperature (RT) for 1 hour followed by overnight incubation with primary antibodies (Zymed Labs, USA) at 4°C. Polyclonal rabbit primary antibodies were used at 1:1000 dilutions for connexin-43(#71-0700), connexin-30 (#71-2200), occludin (#71-1500), claudins 1 and 7 (#51-9000 and 34-9100). Monoclonal mouse primary antibodies were used for Connexin-26 (#33-5800), claudins 2, 4 and 5 (#32-5600, #32-9400, and 35-2500 respectively) at 1 in 1000 dilutions. The next day, blots were washed in 1xTBS-T (0.05% v/v) before incubation with HRP-conjugated goat anti mouse or anti rabbit secondary antibodies at RT for 45 min at 1:10,000 dilution (#115-035-146, #111-035-144; Jackson ImmunoResearch Labs, INC, USA). After three 10 min washes in 1XTBS-T, the blots were immersed in Immobilon Western Chemiluminiscent Horse-Radish Peroxidase substrate solution (Millipore, USA) prepared as per manufacturer's instructions. Images were captured using G-box (Syngene, UK) with Genesnap software at 1, 5 and 10 minutes. Semi-quantification was done using Gene tools gel documentation and analysis software (Syngene, UK). Having first confirmed that the expression of G-βeta was unaffected in Misoprostol treated and untreated pregnant cervical tissue by semi-quantification using western blotting, G-βeta was used as loading control. Human myometrial and placental tissues served as positive controls for GJ and TJ proteins respectively [25,26] and the same protocols were followed as for the cervical tissue.

### Laser scanning confocal microscopy (LSCM)

Localization of the matrix proteins was studied by LSCM imaging by sequential dual indirect immunofluorescence with Cyanine-3 (Cy-3) and Fluorescin isothiocynate-C (FIT-C) conjugated secondary antibodies. 10 μm thick frozen sections were fixed in 100% ice cold acetone for 10 min. After two 5 min washes, blocking of non-specific antigen sites was carried out in 5% goat normal serum (GNS) and 5% bovine serum albumin (BSA) in 1X PBS at RT for 1 hour. Overnight incubations in primary antibodies (all from Zymed labs, USA) were carried out at 4°C as mentioned in section on western blotting: connexin-43, claudins-2, 4, and 5 at 1:100 dilution; 1:200 dilution for connexin-30, occludin, claudins 1 and 7 with 1:60 dilution for connexin-26. Next day, incubations with Cy-3 conjugated goat anti-rabbit antibody (#111-165-144, Jackson ImmunoResearch, USA) and FIT-C conjugated goat anti-mouse secondary antibody (#115-095-146) were carried out sequentially at 1:400 in dark at RT for 45 min each. Negative controls were incubated in either mouse or rabbit isotype control IgG (#08-6599, #08-6199; Zymed Labs, USA) instead of primary antibodies. Positive controls were treated in exactly similar way throughout. After three 10 min washes in 1X TBS-T, sections were air dried before mounting in a drop of Vectashield hard-set mounting medium with DAPI (Vector Labs, USA). Images were acquired with an LSM 510 NLO inverted microscope (Carl Zeiss, Germany) using 20X/0.8 and 40X/1.3 oil immersion objectives. Images were acquired sequentially to eliminate the risk of cross talk. FIT-C was examined using 488 nm Argon laser excitation, a dual band 488/543 dichroic mirror, a NFT545 beam splitter and BP500-530 nm IR emission filter. Cy3 was examined using a 543 nm Helium Neon laser, a dual band 488/543 dichroic mirror, a NFT545 beam splitter and BP565-615 nm IR emission filter. Images of DAPI staining were taken using multiphoton laser tuned to 740 nm a KP650 dichroic mirror and 390-465 nm emission filter. Unstained and control specimens were examined using the same microscope settings as those used to acquire images from the antibody labeled tissue sections.

### Statistical analysis

Data were analysed using SPSS version 18 (Statistical Package for Social Science, Inc., Chicago, IL) employing descriptive statistics, analysis of variance, T-tests, Mann-Whitney U tests and tests of correlation as were appropriate following assessment of normality of distribution (Shapiro-Wilk's test) and equality of variance between groups (the Levene's test), and corrections for multiple comparisons by post hoc analysis. Data was considered significant for p values less than 0.05. LSCM data was not used for quantification.

## Results

All patient samples were studied by both techniques- immune fluorescence analysis and western blotting. The patterns described were seen in all samples and the confocal images and immunoblots shown are representative of the entire samples.

### Effect of Misoprostol on expression of tight junction proteins

**Figure 1:**
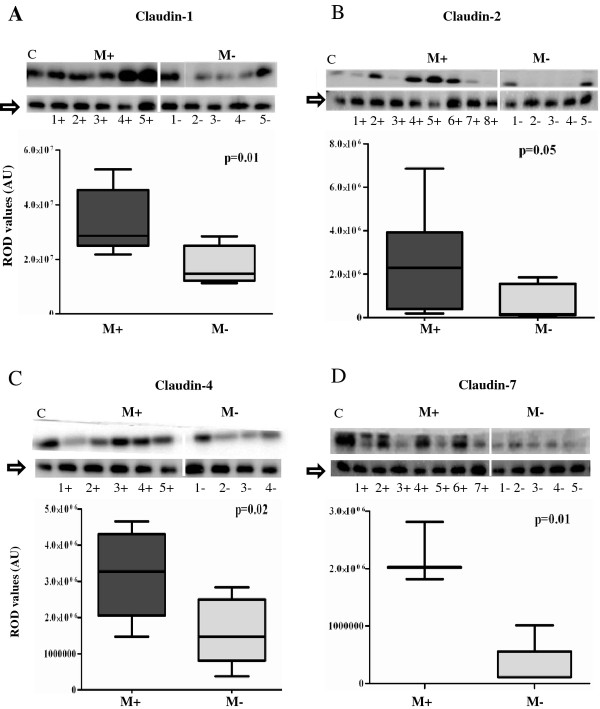
**Comparison of expression levels of claudins- 1 (1A), 2 (1B), 4 (1C) and 7(1D) in Misoprostol treated (M+) (samples 1+ to 7+) versus untreated (M-) (samples 1- to 5-) cervix**. Representative immunoblots in the top panels whilst box plots, lower panel, represent mean (SE) relative optical densities (ROD) for all studied tissues: M+ (n = 10) vs. M- (n = 5). Block arrows showing Gβeta as internal loading control at 35 kDa. C-positive control in the first lane. C-1 = claudin-1; C-2 = claudin-2; C-4 = claudin-4; C-7 = claudin-7. ROD- relative optical density in Arbitrary Units (AU).

**Figure 2:**
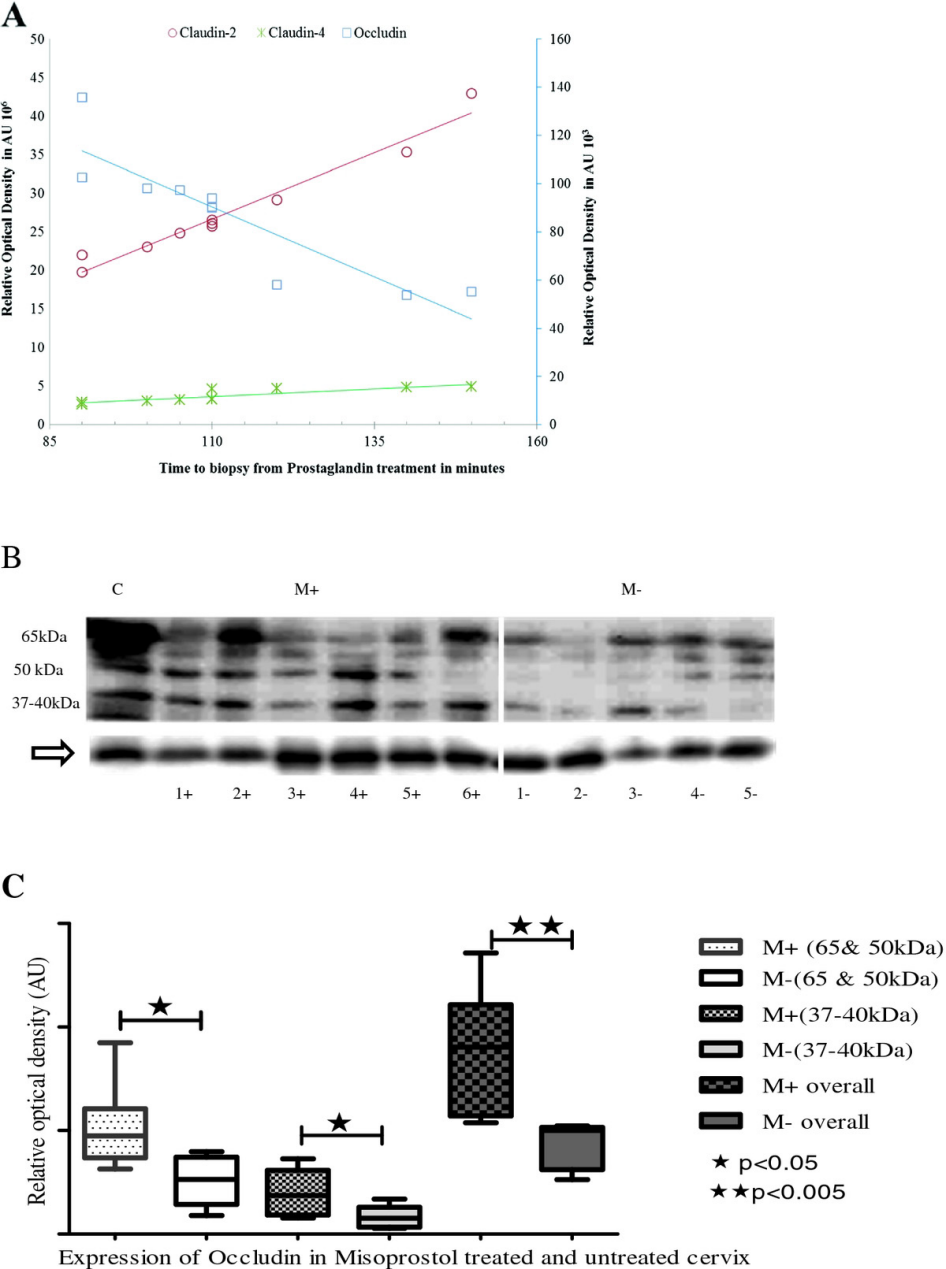
**a: Scatter plot and trend-lines demonstrating positive correlation of claudin-2 (r 0.98, P < 0.001) and claudin-4 (r 0.86, P < 0.05) expressions, and negative correlation of occludin expression (r -0.90, P < 0.05), with time from Misoprostol treatment (M+) to cervical biopsy in minutes**. Occludin relative optical density (ROD) in arbitrary units (AU) is plotted on a secondary y-axis (right) whilst the ROD for claudin-2 and claudin-4 are shown on the primary y-axis (left). **b**: Representative immunoblots depicting significant increase in all isoforms of occludin with Misoprostol treatment (M+) compared to the untreated (M-) cervix in the first trimester of pregnancy. Arrow at G-βeta as internal loading control. C-positive control. **c**: Box plots comparing mean (SE) relative optical densities (ROD) of different isoforms of occludin for all studied tissues: Misoprostol treated (M+, n = 10) vs. Untreated (M-, n = 5). LMW-low molecular weight at 37-40 kDa; HMW- high molecular weight at 50 & 65 kDa; overall- LMW and HMW combined.

There was significantly increased expressions of claudins 1, 2, 4 and 7 (Figure 1a-d) and occludin (Figure 2b, c) in Misoprostol-treated (M+) compared to the untreated (M-) cervix. For occludin, in addition to the 65 kDa bands, we found consistent bands at 50 kDa and between 37-40 kDa in both groups in all specimens studied. Misoprostol-treated tissue demonstrated increased expression of all these bands significantly (p < 0.01 for 50 & 65 kDa and p < 0.05 for 37-40 kDa) (Figure 2b). With increasing time interval from PG treatment to obtaining the biopsy, occludin expression declined (r = -09 P = 0.016), claudins-2 and -4 increased (r = 0.98, P = 0.0001 and r = 0.86, P = 0.014 respectively) whilst Claudin-7 (r = 0.70, P = 0.08) and Claudin-1 (r = -0.07, P = 0.88) expressions did not change significantly. Significant correlations with time of administration of Misoprostol are illustrated in Figure 2a.

### Effects of misoprostol on localisation patterns of tight and gap junction proteins

**Figure 3:**
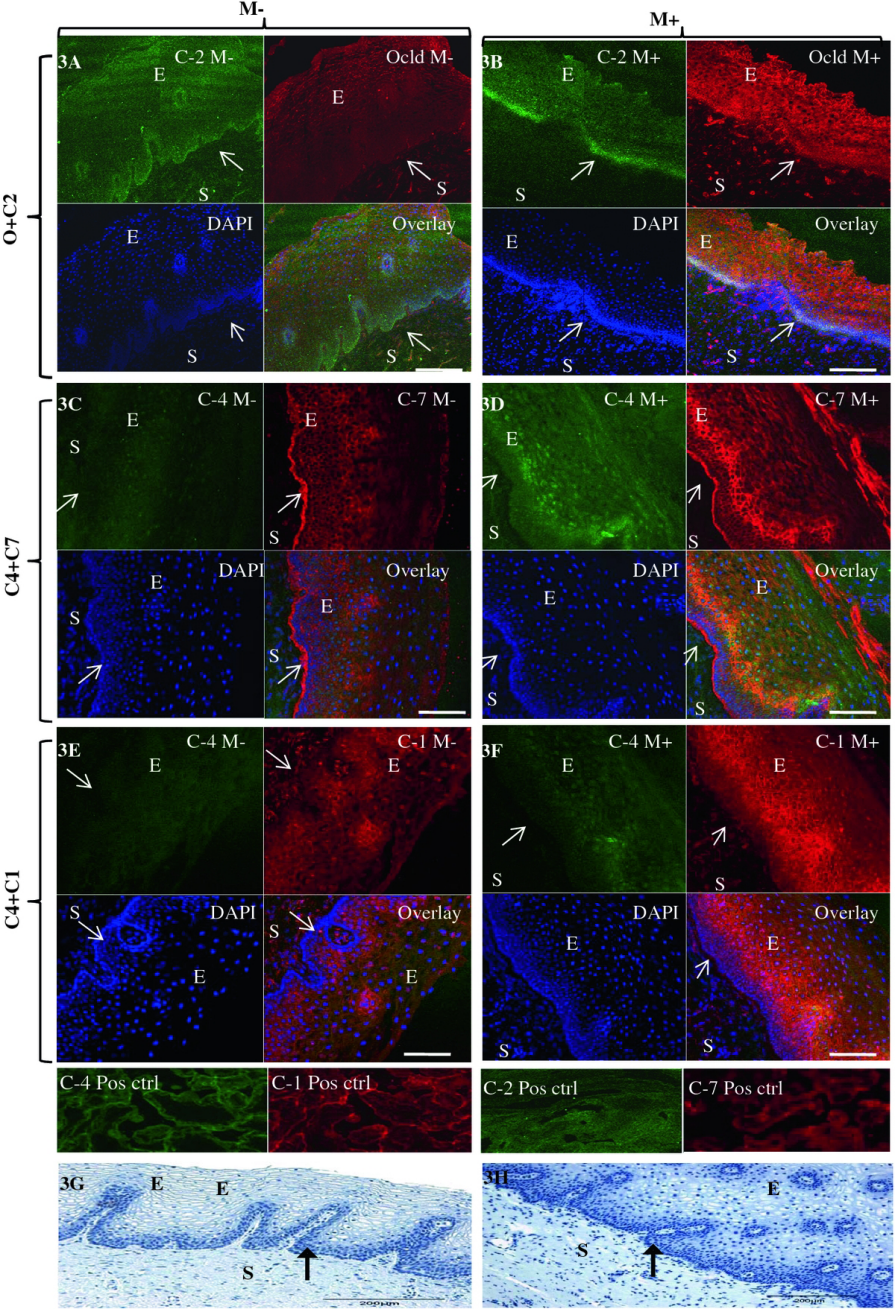
**Representative confocal photomicrographs comparing localization patterns of claudins-1 (C1), -2(C2), -4(C4) and -7(C7) and occludin (Ocld) in Misoprostol treated (M+, right panel) and untreated (M-, left panel) cervix**. The FIT-c (green) and Cyanine-3 (Cy-3-red) labelled secondary antibodies were used; DAPI (blue) for nuclear staining with overlays of red, green and blue (RGB) channels. In the untreated cervix, occludin (red) shifted to intermediate and more superficial layers (3B-red). In the untreated cervix, occludin (3A-red) and claudin-2 (3A green) were expressed in basal layers only (3A-overaly) but following Misoprostol treatment, de novo expression of claudin-2 (3B-green) in the intermediate layer resulted in both now localizing in the basal and intermediate layers (3B-overlay). Compared to diffuse lattice pattern in basal to intermediate layers in the untreated cervix (3C red), Misoprostol induced expression of claudin-7(3D red) in basal and intermediate layers. Claudin-4 was expressed mainly in the nuclei throughout the ectothelium (3C green) in the untreated cervix, with de novo cytoplasmic expression in Misoprostol-treated tissue (3D & 3F green). Claudins 1 and 4 exhibit nuclear and plasma membrane expressions in Misoprostol-treated cohort (3F). Arrows at basement membranes; S-stroma, E-epithelium. 3A- 2 × 2 tile; × 20air objective. NA-0.8, Bar = 8 μm.

Subtle changes in immunolocalisation of TJ in cervical epithelium were observed with Misoprostol treatment: occludin which was mainly expressed in the basal layer of the untreated cervix (Figure 3a-red) appeared to localise more in the intermediate and more superficial layers of tissue treated with Misoprostol (Figure 3b-red). Whereas in the untreated cervix, occludin and claudin-2 were expressed only in the basal layer (Figure 3a-Overlay), Misoprostol treated tissue demonstrated expression of claudin-2 in the intermediate layer (Figure 3b-green), shifting the co-localisation of both proteins to basal and intermediate layers (Figure 3b overlay). Claudin-4 was expressed mainly in nuclei throughout the ectothelium in the untreated cervix (Figure 3c-green), whilst Misoprostol treatment induced expression in the cytoplasmic membranes (Figure 3d, f). Interestingly, claudins 1 and 4 exhibited nuclear and cytoplasmic membrane co-expression in Misoprostol-treated group (Figure 3f) which was absent in the untreated group (Figure 3e). Whilst Claudin-1 was expressed mainly in nuclei throughout the ectothelium in the untreated cervix (Figure 3e-red); PG-treated tissue showed both nuclear and cytoplasmic membrane staining, the latter giving a lattice pattern (Figure 3f). Claudin-7 was expressed in a diffuse lattice pattern in basal and intermediate layers in the untreated cervix (Figure 3c-red), whereas with Misoprostol treatment, expression was mainly in the intermediate layer (Figure 3d-red).

### Vascular expression of claudin-5 and occludin

**Figure 4:**
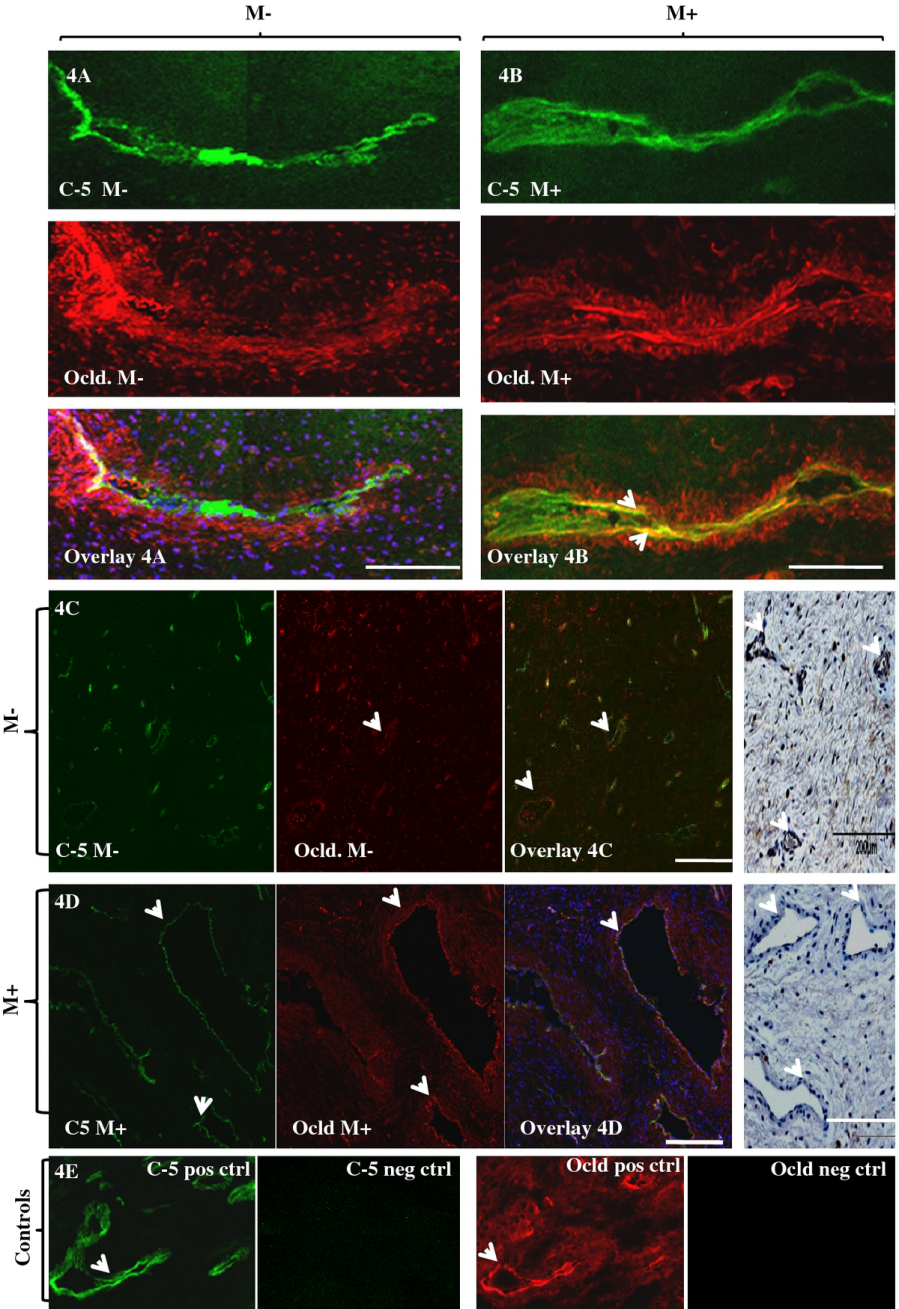
**Effect of Misoprostol treatment on localization of vascular endothelial tight junctions occludin and claudin-5**. **a **and **b**: Representative confocal images comparing expressions of claudin-5 (C-5) (green) and occludin (red) in the cervical vascular endothelium without (4A) and after PG treatment (4B). In the neat (PG-) cervix, C-5 was selectively localized in the endothelium (4A-green) whilst occludin (Ocld) was mainly expressed in the smooth muscle (4A-red). Treatment with misoprostol (M+) induced expressions of both C-5 and occludin (4B) compared to the untreated (M-) cervix (4A). Also, de novo endothelial expression of occludin was seen with PG treatment (4B red vs. 4A red; overlays 4A vs. 4B). × 40, oil immersion; NA = 1.3; Bar 8 μm. **c**-**d**: Representative confocal photomicrographs showing larger calibre of blood vessels in the PG treated (PG+) compared to the untreated (PG-) cervix (Figure 4D Vs. 4C) ×20 air objective, NA 0.8, Bar = 8 μm. Extreme right panels depicting H & E stained histological section of cervical stroma in the M+ and M- tissues. E: Positive and negative controls for occludin and claudin-5.

Claudin-5 was selectively localized to the vascular endothelium (Figure 4a-green) whilst occludin was expressed in the vascular smooth muscle in addition to the squamous ectothelium in the human cervix (Figure 4a-red). PG treatment appeared to increase expression and also cause co-localization of claudin-5 and occludin in the vascular endothelium (Figure 4b-overlay). Furthermore, the blood vessels in the PG-treated cervix were consistently larger compared to the un-treated cervix (Figure 4d vs. 4c). Our attempts at immuno-blotting of claudin-5 were unsuccessful.

### Effect of Misoprostol treatment on expression of Gap junction proteins

**Figure 5:**
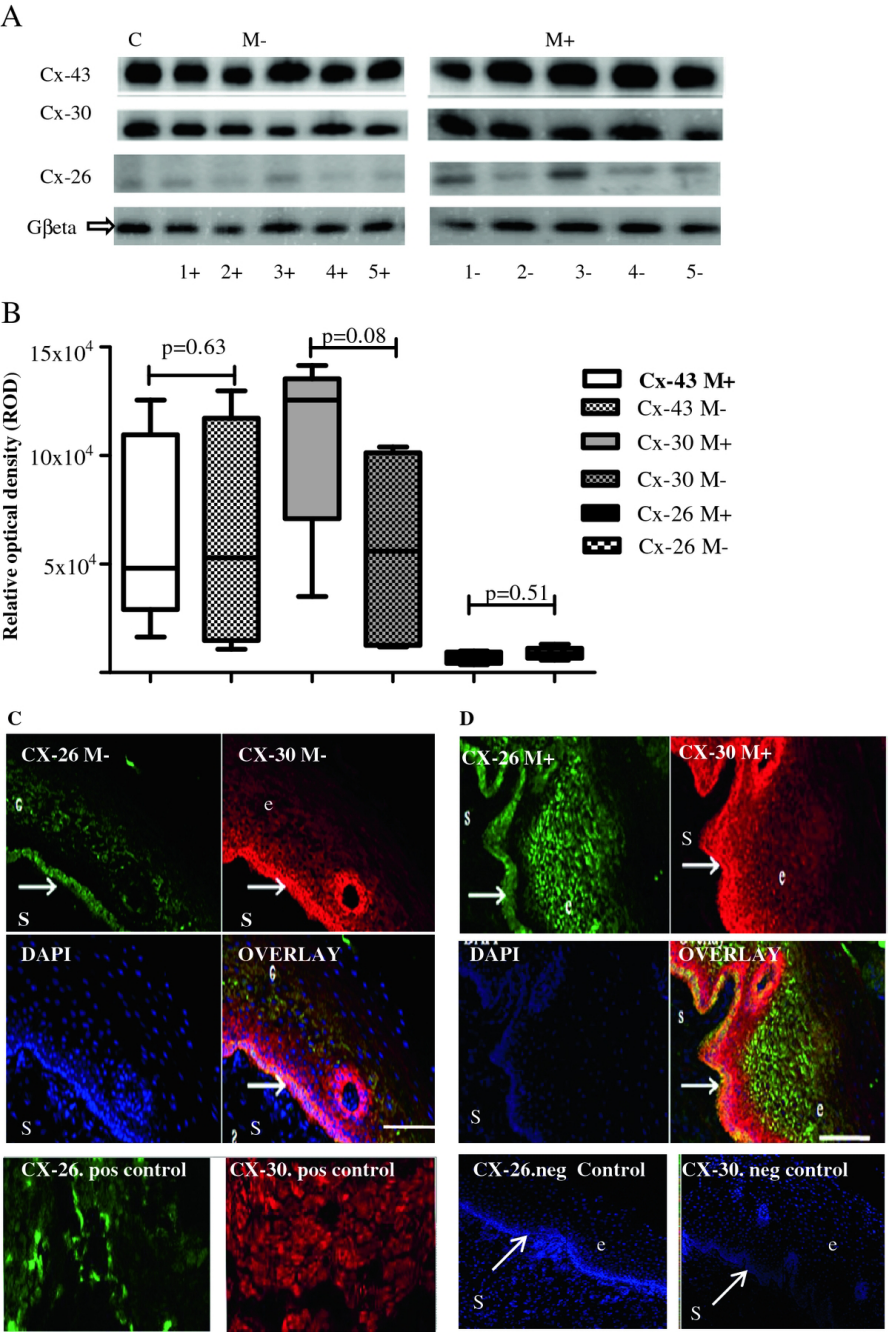
**Effect of prostaglandin treatment on expression of gap junction proteins-connexins 43, 30 and 26**. A: Representative immunoblots of connexins 43, 30 and 26 in untreated (M-) and Misoprostol treated (M+) cervix in first trimester. C-positive control. B: Graphical representation comparing expressions of connexin- 43 (CX-43), connexin-30 (CX-30) and connexin-26 (CX-26) in Misoprostol treated (M+, n = 10) versus untreated (M-, n = 5) cervix on Western blotting. The expression of the studied connexions did not differ between Misoprostol-treated and untreated tissue. C and D: Representative confocal photomicrographs depicting no change in localisation patterns of connexins 30 and 26 in untreated (M-) (Figure 5C) versus Misoprostol treated (M+) (Figure 5D) cervix. Bottom row showing positive controls (myometrium) and negative controls (cervical tissue) for connexins 26 and 30. DAPI- nuclear staining. e- epithelium, s-stroma. Arrows at basement membranes. ×20 air objective, NA 0.8, Bar = 8 μm.

Misoprostol treatment was not associated with significantly altered expression of any of the GJ proteins studied: a trend towards increased expression of Cx-30 in Misoprostol-treated tissue did not attain statistical significance (Figure 5a and 5b). Furthermore, immune-localisation patterns of these GJs in human cervical ectothelium did not differ with Misoprostol treatment (Figure 5c and 5d).

## Discussion

This study reports, for the first time, that cervical remodeling induced by Misoprostol in the first trimester is associated with increased expression of claudins 1, 2, 4, 7 and occludin, as well as increased vascular endothelial expression of claudin-5 with concomitant vasodilatation. Our findings support the hypothesis that cervical TJs may play an important role in Misoprostol-induced cervical remodelling. Taken together with recent reports that PG-induced cervical remodelling involves increased stromal leukocytes and cytokines [27] which can regulate paracellular permeability [28], our observations strongly suggest that altered TJ expression and function may modulate cervical ECM remodeling by altering paracellular permeability.

We found a positive correlation between expression levels of claudins 2 and 4 and the time interval from Misoprostol treatment to biopsy, further supportive of the thesis that Misoprostol administration and TJ expression are linked, perhaps causally. It is tempting to speculate that this temporal change in expression levels may be attributable to changes associated with the signaling cascade involved in cervical remodelling. Clinical trials have shown that the efficacy of vaginal Misoprostol for cervical priming is both dose- and time-dependent [11,14], and is related to time to attain peak plasma concentrations (70-80 min after vaginal administration) [29]. It is plausible that the increase in cervical epithelial TJ expression in response to Misoprostol treatment serves to promote trans-epithelial permeability, increasing local tissue delivery of Misoprostol and its plasma concentrations. Further studies are required to confirm this, and whether TJs play a more direct modulatory role in CR.

Misoprostol significantly increased expression of all the forms of occludin: we consistently found occludin bands at 65 kDa, 50 kDa and between 37-40 kDa in all studied specimens. Previous in vitro studies have attributed such bands between 62 to 82 kDa on SDS-PAGE to phosphorylated forms of occludin [30-32]. Whether post-translational modification of occludin by phosphorylation is a mechanism by which Misoprostol treatment alters trans-epithelial resistance and permeability during cervical remodelling is unclear. An alternative hypothesis has been that the 50 kDa and 37-40 kDa isoforms of occludin may reflect rapid occludin turnover during pregnancy [33]. Such breakdown turnover to the 50 kDa isoform has been shown to reduce TJ resistance of cervical epithelium [34], suggesting that low molecular weight forms of occludin, being low resistance pathways, may increase epithelial layer permeability leading to increased stromal hydration. The significance of the inverse correlation of cervical occludin with time of Misoprostol administration is unclear. One explanation is that the maximal effect of Misoprostol on occludin expression is achieved very rapidly compared to claudins 2 and 4, waning thereafter in a time-dependent fashion.

Claudin-5 was selectively localized to the vascular endothelium whilst occludin was expressed mainly in the vascular smooth muscle. Their increased expression and mediation of trans-endothelial migration of T lymphocytes and macrophages [35], together with the reported recruitment by PG of chemotactic leukocytes [16] and mast cells [6], are strongly supportive of important roles for TJ in the regulation of vascular permeability [15,27,36] in the ECM during Misoprostol-induced CR.

Whether Misoprostol-induced nuclear membrane expression of claudins 1 and 4 (Figure 3f) observed by us could imply increased transcription or interaction with nuclear PGE_1-4 _receptors as suggested by others [37] needs further investigation. Misoprostol did not significantly affect the expression of gap junction proteins, suggesting that they are unlikely to play key roles in Misoprostol-induced CR.

The interpretation of our observations should be subject to some caveats. Firstly, we have not demonstrated any significant effect of misoprostol on cervical GJ expression in the limited number of tissue samples studied. However ethical constraints precluded taking further biopsy samples once significant effects had already been demonstrated for TJ expression. We would have needed more than two hundred cervical biopsies to demonstrate a 20% difference in the expression of connexins- 43 and 26 between Misoprostol-treated and non-treated cervix with 80% power at the 95% confidence level. It is plausible that a role for GJs in cervical ripening was not detectable by our limited sample sizes and assay techniques, or that GJs may modulate remodelling processes induced by other synthetic or naturally-occurring prostagladin analogues which we have not studied. Secondly, the heterogeneity of expression of different TJs (seen as varying band intensities in western blotting- Figures 1 and 2b) in the Misoprostol-treated group could be partly due to the varied duration of exposure to Misoprostol, as well as individual variations in subject responses [27]. Despite these considerations we still observed significant differences between Misoprostol-treated and untreated cervix, strongly suggesting a true effect of Misoprostol on cervical expression of some TJ proteins. It was not practicable to fix the time interval between administration of Misoprostol and the cervical biopsy as this depended on a highly variable transit time between the wards, the operating theatres and the administration of anaesthetic for the clinical procedure. Recording the variation in time to biopsy from Misoprostol administration enabled us to serendipitously observe a time-dependent change in expression of some of the TJ proteins studied.

## Conclusion

We report for the first time that cervical tissue treated with Misoprostol, a synthetic PGE1 analogue, demonstrated increased expression of specific epithelial and endothelial TJ proteins in the pregnant human cervix in a time-dependent fashion. This suggests that signals that regulate trans-epithelial transport in the cervix may act by regulating TJ mediated paracellular permeability, highlighting a potential role for these matrix proteins in modulating cervical remodelling induced by Misoprostol. Further studies are needed to clarify the mechanism of this interaction during tissue remodelling, and to determine whether our observations in first trimester are mirrored during spontaneous ripening at term. These studies are necessary as they may clarify the mechanism of failed initiation of labour using PG analogues, as well as uncover new therapeutic targets.

## Competing interests

The authors declare that they have no competing interests.

## Authors' contributions

VG carried out all the laboratory experiments, participated in the data analysis and drafted the manuscript. CG supervised the confocal microscopy experiments. AG participated in the recruitment of participants. DA conceived of the study, and participated in its design and coordination and helped to draft the manuscript. All authors read and approved the final manuscript.

## References

[B1] Olah KS The use of Magnetic Resonance Imaging in the assessment of cervical hydration state Br J Obstet Gynecol 1994 101 255 257 10.1111/j.1471-0528.1994.tb13122.x 8193104

[B2] Leppert PC Anatomy and physiology of cervical ripening Clin Obstet Gynecol 1995 38 267 279 10.1097/00003081-199506000-00009 7554594

[B3] Munoz-de-Toro M Varayoud J Ramos JG Rodriguez HA Luque EH Collagen remodelling during cervical ripening is a key event for successful vaginal delivery Braz J Morphol Sci 2003 20 75 84

[B4] Uldberg N Ekman G Malmstrom A Olsson K Ulmsten U Ripening of human uterine cervix related to changes in collagen, glycosaminoglycans and collagenolytic activity Am J Obstet Gynecol 1983 147 662 666 6638110 10.1016/0002-9378(83)90446-5

[B5] Ekerhovd E Brannstrom M Weijdegard B Norstrom A Nitric oxide synthases in the human cervix at term pregnancy and effects of nitric oxide on cervical smooth muscle contractility Am J Obstet Gynecol 2000 183 610 616 10.1067/mob.2000.105901 10992181

[B6] Radulovic N Ekerhovd E Abrahamsson G Norstrom A Cervical priming in the first trimester: morphological and biochemical effects of misoprostol and isosorbide mononitrate Acta Obstet Gynecol Scand 2009 88 43 51 10.1080/00016340802585440 19034732

[B7] Malmstrom E Sennstrom M Holmberg A Frielingsdorf H Eklund E Malmstrom L Tufvesson E Gomez MF Westergren-Thorsson G Ekman-Ordeberg G The importance of fibroblasts in remodelling of the human uterine cervix during pregnancy and parturition Mol Hum Reprod 2007 13 333 341 10.1093/molehr/gal117 17337476

[B8] Stjernholm-Vladic Y Stygar D Mansson C Masironi B Akerberg S Wang H Ekman G Sahlin L Factors involved in the inflammatory events of cervical ripening in humans Reprod Biol Endocrinol 2004 2 74 10.1186/1477-7827-2-74 15500686 PMC534613

[B9] Hertelendy F Zakár T Prostaglandins and the myometrium and the cervix Prostaglandins Leukot Essent Fatty Acids 2004 70 207 222 10.1016/j.plefa.2003.04.009 14683694

[B10] Anderson J Brown N Mahendroo MS Reese J Utilization of different aquaporin water channels in the mouse cervix during pregnancy and parturition and in models of preterm and delayed cervical ripening Endocrinology 2006 147 130 140 16179408 10.1210/en.2005-0896

[B11] Singh K Fong YF Preparation of the cervix for surgical termination of pregnancy in the first trimester Hum Reprod Update 2000 6 442 448 10.1093/humupd/6.5.442 11045875

[B12] Shultz KF Grimes DA Cates WJ Measures to prevent cervical injury during suction curettage abortion Lancet 1983 1182 1184 6133988 10.1016/s0140-6736(83)92464-9

[B13] Radulovic N Norström A Ekerhovd E Outpatient cervical ripening before first-trimester surgical abortion: a comparison between Misoprostol and isosorbide mononitrate Acta Obstet et Gynecol Scand 2007 86 344 348 10.1080/00016340601134598 17364311

[B14] Singh K Fong YF Prasad RNV Dong F Minimal evacuation interval after vaginal misoprostol for pre-abortion cervical priming: a randomized trial Obstet Gynecol 94 431 434 10472873 10.1016/s0029-7844(99)00272-0

[B15] Kelly RW Inflammatory mediators and cervical ripening J Reprod Immunol 2002 57 217 224 10.1016/S0165-0378(02)00007-4 12385844

[B16] Kelly RW Pregnancy maintenance and parturition: the role of prostaglandin in manipulating the immune and inflammatory response Endocr Rv 1994 15 684 706 10.1210/edrv-15-5-684 7843072

[B17] Zeng R Li X Gorodeski GI Estrogen abrogates transcervical tight junctional resistance by acceleration of occludin modulation J Clin Endocrinol Metab 2004 89 5145 5155 10.1210/jc.2004-0823 15472219

[B18] Sobel G Páska C Szabò I Kiss A Kádár A Schaff Z Increased expression of claudins in cervical squamous intraepithelial neoplasia and invasive carcinoma Hum Pathol 2005 36 162 169 10.1016/j.humpath.2004.12.001 15754293

[B19] Sobel G Szabò I Páska C Kiss A Kovalszky I Kádár A Paulin F Schaff Z Changes of cell adhesion and extracellular matrix (ECM) components in cervical intraepithelial neoplasia Pathol Oncol Res 2005 11 26 31 10.1007/BF03032402 15800679

[B20] Timmons BC Mitchell SM Gilpin C Mahendroo MS Dynamic changes in the cervical epithelial tight junction complex and differentiation occur during cervical ripening and parturition Endocrinol 2007 148 1278 1287 10.1210/en.2006-0851 17138657

[B21] Tsukita S Furuse M Itoh M Multifunctional strands in tight junctions Nat Rev Mol Cell Biol 2001 2 285 290 10.1038/35067088 11283726

[B22] Laird DW Life cycle of connexins in health and disease Biochem J 2006 394 527 543 16492141 10.1042/BJ20051922 PMC1383703

[B23] Aasen T Graham SV Edward M Hodgins MB Reduced expression of multiple gap junction proteins is a feature of cervical dysplasia Mol Cancer 2005 4 31 10.1186/1476-4598-4-31 16091133 PMC1198252

[B24] Saito Y Maki M Sakamoto H Gap junction formation in the human uterine muscle cell of the corpus and cervix during the menstrual cycle and pregnancy Nippon Sanka Fujinka Gakkai Zasshi 1987 39 135 140 3819505

[B25] Lievano S Alarcon L Chavez-Munguia B Gonzalez-Mariscal L Endothelia of term human placentae display diminished expression of tight junction proteins during pre-eclampsia Cell Tissue Res 2006 324 433 448 10.1007/s00441-005-0135-7 16508790

[B26] Chow L Lye SJ Expression of the gap junction protein connexin-43 is increased in the human myometrium toward term and with the onset of labor Am J Obstet Gynecol 1994 170 788 795 8141203 10.1016/s0002-9378(94)70284-5

[B27] Sahlin L Stjernholm-Vladic Y Roos N Masironi B Ekman-Ordeberg G Impaired leukocyte influx in cervix of post-term women not responding to prostaglandin priming Reprod Biol Endocrinol 2008 6 36 10.1186/1477-7827-6-36 18764934 PMC2551600

[B28] Nusrat A Turner JR Madara JL Molecular physiology and pathophysiology of tight junctions: IV. Regulation of tight junctions by extracellular stimuli: nutrients, cytokines and immune cells Am J Physiol Gastrointest Liver Physiol 2000 279 G851 G857 11052980 10.1152/ajpgi.2000.279.5.G851

[B29] Tang OS Schweer H Seyberth HW Lee SWH Ho PC Pharmacokinetics of different routes of administration of Misoprostol Hum Reprod 2002 17 332 336 10.1093/humrep/17.2.332 11821273

[B30] González-Mariscal L Tapia R Chamorro D Cross talk of tight junction components with signaling pathways Biochemica et Biophysica Acta 2008 1778 729 756 10.1016/j.bbamem.2007.08.018 17950242

[B31] Farshori P Kachar B Redistribution and phosphorylation of occludin during opening and resealing of tight junctions in cultured epithelial cells J Membr Biol 1999 170 147 156 10.1007/s002329900544 10430658

[B32] Sakakibara A Furuse M Saitou M Ando-Akatsuka Y Tsukita S Possible involvement of phosphorylation of occludin in tight junction formation J Cell Biol 1997 137 1393 1401 10.1083/jcb.137.6.1393 9182670 PMC2132539

[B33] Wong V Phosphorylation of occludin correlates with occludin localization and function at the tight junction Am J Physiol Cell Physiol 1997 273 C1859 C1867 10.1152/ajpcell.1997.273.6.C1859 9435490

[B34] Zhu L Li X Zeng R Gorodeski GI Changes in tight junction resistance of the cervical epithelium are associated with modulation of content and phosphorylation of occludin 65-kilodalton and 50- kilodalton forms Endocrinol 2006 147 977 989 10.1210/en.2005-0916 PMC2409057 16239297

[B35] Martin-Padura I Lostaglio S Schneemann M Williams L Romano M Fruscella P Panzeri C Stoppacciaro A Ruco L Villa A Junctional adhesion molecule, a novel member of the immunoglobulin superfamily that distributes at intercellular junctions and modulates monocyte transmigration J Cell Biol 1998 142 117 127 10.1083/jcb.142.1.117 9660867 PMC2133024

[B36] Osman I Young A Ledingham MA Thomson AJ Jordan F Greer IA Norman JE Leukocyte density and pro-inflammatory cytokine expression in human fetal membranes, decidua, cervix and myometrium before and during labour at term Mol Hum Reprod 2003 9 41 45 10.1093/molehr/gag001 12529419

[B37] Bhattacharya M Peri K Da-Silva R Localization of functional prostaglandin E2receptors, EP3 and EP4 in the nuclear envelope J Biol Chem 1999 274 15719 15724 10.1074/jbc.274.22.15719 10336471

